# Characterization of *constricted fruit (ctf)* Mutant Uncovers a Role for *AtMYB117/LOF1* in Ovule and Fruit Development in *Arabidopsis thaliana*

**DOI:** 10.1371/journal.pone.0018760

**Published:** 2011-04-13

**Authors:** Maria Dolores Gomez, Cristina Urbez, Miguel A. Perez-Amador, Juan Carbonell

**Affiliations:** Instituto de Biología Molecular y Celular de Plantas, CSIC-Universidad Politécnica de Valencia, Valencia, Spain; University of Melbourne, Australia

## Abstract

Pistil and fruit morphogenesis is the result of a complex gene network that is not yet fully understood. A search for novel genes is needed to make a more comprehensive model of pistil and fruit development. Screening for mutants with alterations in fruit morphology generated by an activation tagging strategy resulted in the isolation of the *ctf* (constricted fruit) mutant. It is characterized by a) small and wrinkled fruits, with an enlarged replum, an amorphous structure of the septum and an irregular distribution of ovules and seeds; b) ectopic carpelloid structures in sepals bearing ovule-like structures and c) dwarf plants with curled rosette leaves. The overexpressed gene in *ctf* was *AtMYB117*, also named *LOF1 (LATERAL ORGAN FUSION1)*. *AtMYB117/LOF1* transcripts were localized in boundary regions of the vegetative shoot apical meristem and leaf primordia and in a group of cells in the adaxial base of petioles and bracts. Transcripts were also detected in the boundaries between each of the four floral whorls and during pistil development in the inner of the medial ridges, the placenta, the base of the ovule primordia, the epidermis of the developing septum and the outer cell layers of the ovule funiculi. Analysis of changes of expression of pistil-related genes in the *ctf* mutant showed an enhancement of *SHATTERPROOF1* (*SHP1*) and *SHP2* expression. All these results suggest that *AtMYB117/LOF1* is recruited by a variety of developmental programs for the establishment of boundary regions, including the development of floral organs and the initiation of ovule outgrowth.

## Introduction

Meristem activity determines plant development. All above-ground organs, vegetative and reproductive, originate from the shoot apical meristem (SAM). When plants initiate flowering, the vegetative SAM is transformed into an inflorescence meristem (IM). The IM, in turn, generates a collection of undifferentiated cells called floral meristems that give rise to flowers. The flower contains reproductive structures, such as stamens and pistils that enclose the ovules which develop into seeds upon fertilization. Therefore, the fruit with mature seeds is the final product of a developmental process initiated in floral meristems and ovule primordia [1], [2]. The transition of meristem into meristem or differentiated tissue involves the formation of boundary layers of cells, also present between adjacent organs, with characteristics of both meristem and fully differentiated cells, such as reduced growth activity. These boundaries are considered reference points for the generation of new meristems and lateral organ primordia [3].

*Arabidopsis* fruit are siliques that originate from pistils constituted of two fused carpels, which are essentially modified leaves [4]. Pistils are composed of the ovary (with ovules and multiple tissues: replum, septum, valves and valve margin), the style and the stigma at the top. Twenty developmental stages have been proposed between the formation of the flower buttress and silique dehiscence, providing common landmarks to describe developmental events [4]. Flower primordia form at stage 2 while the onset of pistil development occurs at stage 6. At stage 7 the pistil grows as a hollow tube. At stage 8, early repla give rise to two medial ridges flanked on both sides by placental tissues. At stage 9, the septum forms when the medial ridges fuse and the placenta produces ovule primordia. The ability to generate differentiated tissues indicates that the early repla possess meristematic characteristics. This meristematic zone does not correspond to a true meristem but a zone of active cell proliferation within the organ that gives rise to specialized and determinate tissues, such as all the marginal tissues of the fruit (replum, septum, ovules, style and stigma), and has therefore been termed a “quasi meristem” [1]. The valves and valve margins are the only fruit tissues that develop independently of this meristematic activity. At stage 13, flowers reach anthesis and stigma are pollinated. After fertilization, at stage 14, the pistils are transformed into developing fruit.

Several genes implicated in pistil and fruit development have been identified. Some genes regulate development and tissue patterning in both SAM and fruit marginal tissues, or in both leaf and fruit lateral tissues, suggesting a common origin of these organs [1], [4], [5]. While previously identified genes are certainly important components of flower development, a search for novel genes is needed to form a more comprehensive model of pistil and fruit development.

Identifying novel genes involved developmental processes often requires bypassing the functional redundancy of genes constituting a family. In this regard, an activation tagging strategy that consists of the insertion of the *Cauliflower Mosaic Virus* (CaMV) 35S enhancers, that act differently than the complete CaMV 35S promoter, has been selected [6]. The activation tagging strategy has been successfully used [7] as mutant phenotypes are caused by overexpression rather than knock-out of the targeted gene. Therefore, we used this strategy to search for genes involved in pistil and fruit development in *Arabidopsis*. We selected a dominant mutant showing altered fruit morphology that we named *ctf* for *constricted fruit*. The overexpressed gene in *ctf* was *AtMYB117*, a member of the MYB family of transcription factors [8], [9]. *AtMYB117* was recently described as *LOF1* (*LATERAL ORGAN FUSION1*), functioning in organ boundary specification, meristem initiation and organ patterning [10]. GUS activity in an enhancer-trap line suggests that *AtMYB117/LOF1* expression is localized to organ boundaries [10]. Additionally, in *lof1-1* lines, a T-DNA mutant with a partial phenotype, *AtMYB117/LOF1* expression was undetectable in both pedicel nodes and paraclade junctions, in conjunction with visible defects, but was only slightly reduced in inflorescence apex where no phenotypic defects were observed. These results [10], and the alterations in the morphology of the fruit in *ctf* prompted us to examine in more detail the expression of *AtMYB117/LOF1* using *in situ* hybridization. In addition, we generated an artificial microRNA (amiRNA) [11] to silence *AtMYB117/LOF1* and *AtMYB105/LOF2* (closely related to *AtMYB117/LOF1*) expression to study a possible role for *AtMYB117/LOF1* in the development of reproductive organs.

## Results

### Isolation and morphological characterization of *ctf* mutants

**Figure 1:**
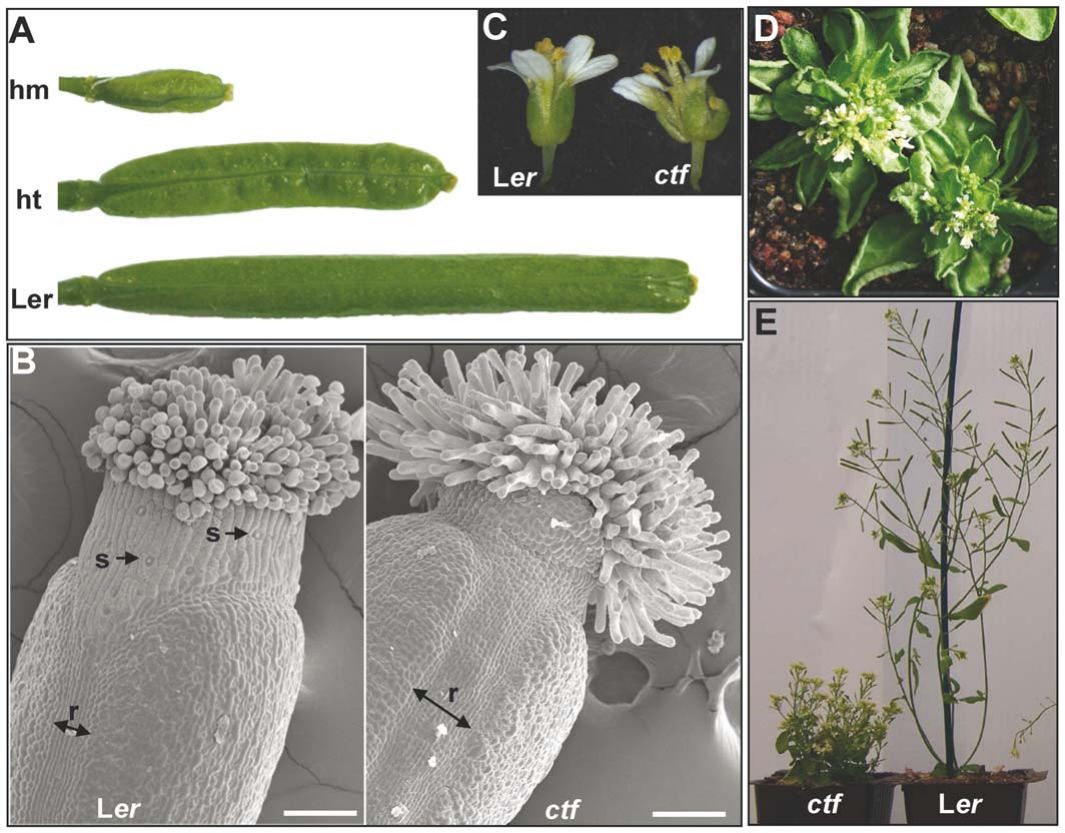
Phenotype of the *ctf* mutant. (**A**) Fruits from wild type L*er* and heterozygous (ht) or homozygous (hm) *ctf* mutants. (**B**) Scanning electron micrograph of L*er* and *ctf* mutant pistil at floral stage 12. (**C**) Flower from L*er* and *ctf* mutant. (**D**) *ctf* plants showing upward curled rosette leaves. (**E**) Wild type (right) and *ctf* mutant (left) plants. *ctf* and L*er* plants were 8 weeks old. r, replum; s, stoma. Scale bars are 100 µm.

To isolate *Arabidopsis* mutants with alterations in pistil and fruit development we screened approximately 5000 activation-tagged primary lines and identified a mutant which was named *ctf*, according to its morphological characteristics, that is, a small and wrinkled fruit (Figure 1A). T_2_ plants segregated for the constricted fruit characteristic in a 1∶2∶1 ratio, suggesting a single insertion locus. The fruit phenotype initially observed in heterozygous plants became more severe in homozygous individuals, indicating that the phenotype is determined by a semidominant allele (Figure 1A), which is consistent with the overexpression of a gene caused by CaMV 35S enhancers. Other remarkable characteristics of homozygous *ctf* fruits were that the replum was wider and the stigmatic papillae were more elongated (Figure 1B). The cells of *ctf* style did not have the wax crenulations present in the wild type style cells, and *ctf* style was composed of small and smooth cells that did not have visible stomata (Figure 1B). *ctf* plants also showed alterations in leaf, inflorescence and flower development. Flowers had an altered morphology with abnormal separated organs and upward curled leaves in the rosette (Figures 1C and D). In addition, *ctf* plants were smaller than wild type with short internodes and bushy appearance due to the development of multiple stems and loss of apical dominance (Figure 1E).

**Figure 2:**
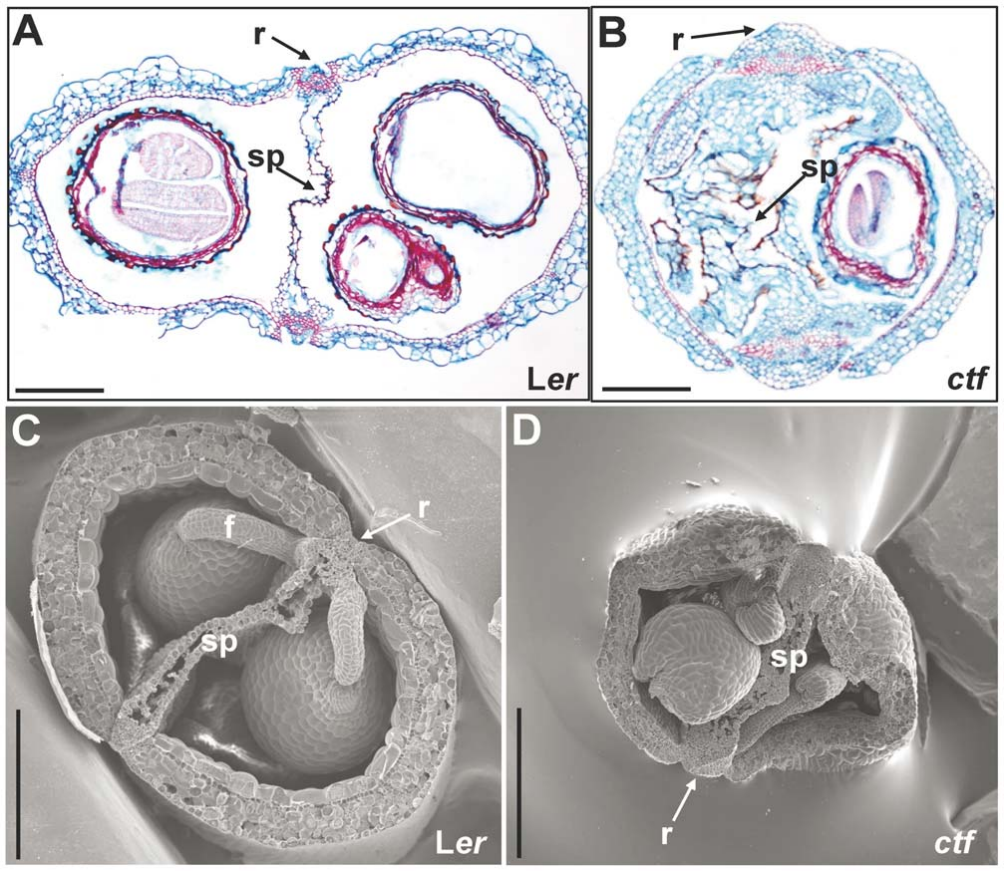
Histological analysis of fruits in *ctf*. Paraffin cross sections of L*er* (**A**) and *ctf* (**B**) fruits stained with Alcian blue and Safranin O at late stage 17. Cryo-scanning electron micrographs of transverse sections of L*er* (**C**) and *ctf* (**D**) fruits at early stage 17. f, funiculus; r, replum; sp, septum. Scale bars are 200 µm in **A** and **B**, and 300 µm in **C** and **D**.

**Figure 3:**
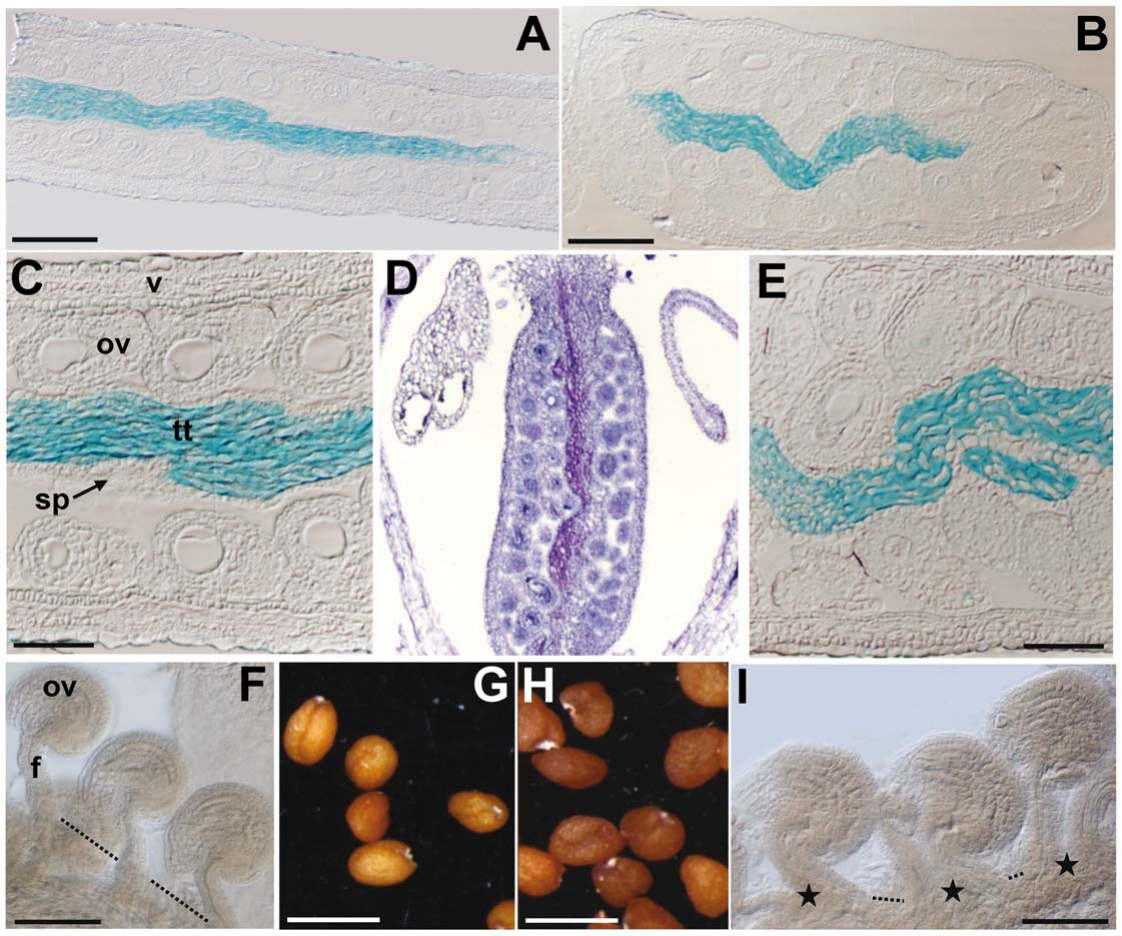
Morphological characteristics of ovules and seeds in *ctf*. Resin longitudinal sections of a L*er* (**A**) and *ctf* pistil (**B**) in anthesis. The transmitting tissue was stained with Alcian blue. Close-up view of L*er* (**C**) and *ctf* (**E**) ovules, septum and transmitting tissue. Paraffin longitudinal section of *ctf* pistil in anthesis stained with Toluidine blue (**D**). Whole-mount cleared ovules and funiculi of a L*er* (**F**) and *ctf* (**I**) pistil in anthesis. Note the distance between funiculi (stars indicate close funiculi and dotted lines mark the distance between separated funiculi). L*er* (**G**) and *ctf* (**H**) seeds. f, funiculus; ov, ovule; sp, septum; tt, transmitting tissue; v, valve. Scale bars are 200 µm in **A**, **B**, **F** and **I**; 100 µm in **C** and **E**; and 500 µm in **G** and **H**.

Transverse sections of homozygous *ctf* fruit revealed an irregular distribution of seeds, an enlarged replum and an amorphous structure of the septum (Figures 2A–D). Frequently, the irregular septum filled almost the whole cavity of the fruit (Figure 2B). Longitudinal sections of Landsberg *erecta* (L*er*) pistils showed the septum in the centre and a row of ovules in each locule (Figures 3A and C). In contrast, the ovules in *ctf* pistils were not organized in rows (Figures 3B, D and E), but were piled up on each other, which caused a distortion in the septum linearity. Whole-mount cleared ovaries were examined to compare the morphology of L*er* and *ctf* ovules. Compared to wild type, the ovules in *ctf* ovaries developed closer to each other. The distance between *ctf* funiculi was smaller than between L*er* funiculi (Figures 3F and I), and the ovules were forced to be distributed at various levels, as they did not fit in the same plane, possibly explaining why a row of ovules per carpel was not observed in *ctf* sections. In addition, ovules and seeds in *ctf* mutant were slightly larger than those in L*er* plants (Figures 3F–I).

**Figure 4:**
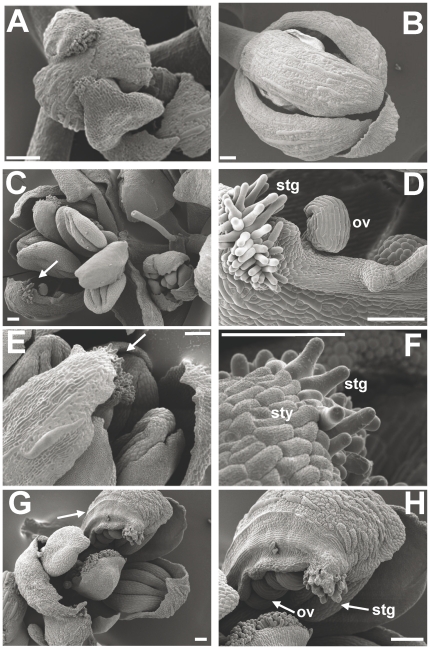
Cryo-scanning electron micrographs of *ctf* (A, C–H) and L*er* (B) flowers. *ctf* flowers showing carpelloid sepals (arrows) (**C**, **E**, **G**), and close-ups of the carpelloid sepals (**D**, **F**, **H**). ov, ovule; stg, stigma; sty, style. Scale bars are 100 µm.

To thoroughly study the *ctf* flower morphology, *ctf* inflorescences were observed by cryo-scanning electron microscopy (cryo-SEM). *ctf* flowers in anthesis from 6-week old plants generated ectopic carpelloid structures (Figures 4A, C, E, G) that were not visible in flowers of L*er* and younger *ctf* plants (Figure 4B). Stigmatic papillae (Figures 4D, F, H) and style-like regions (Figure 4F) developed on the edges of the sepals. The cells in these regions showed typical wax crenulations of style cells. The carpelloid sepals often appeared folded and, on the inner side, ovule-like structures developed (Figures 4D and H). Seventy-three percent (11/15) of analyzed *ctf* plants exhibited flowers with carpelloid sepals, while wild type L*er* plants did not show this phenotype (data not shown).

To better characterize the carpelloid structures observed in sepals of *ctf* flowers, we searched for the presence of *SPATULA* (*SPT*) transcripts. *SPT* is a transcription factor that promotes growth of tissues arising from the carpel margins, including ovules, septum and transmitting tract, as well as style and stigma [12]. *ctf* plants were crossed with L*er* plants carrying *pSPT-6253:GUS*, the *SPT* promoter region fused to a GUS reporter gene [13]. In *ctf* sepals, GUS expression was recorded in stigmatic papillae, ovules and in the edges of the sepals where the ovules arise (Figures S1A and B). These blue edges consisted of typical cells of transmitting tissue (Figure S1C). These cells could not be identified in the cryo-SEM images.

### Molecular characterization of *ctf* mutants

Plasmid rescue revealed that the T-DNA containing the CaMV 35S enhancer in *ctf* plants was inserted into chromosome 1 between *At1g26770* and *At1g26780*, the two immediately adjacent genes that are transcribed in the same orientation (Figure S2). The expression of *At1g26770* and *At1g26780* and three other proximal genes, *At1g26760*, *At1g26790* and *At1g26795*, was analyzed by qRT-PCR. Of the five genes, only the expression of *At1g26780* was highly increased in *ctf* mutants relative to wild type plants. *At1g26780* encodes the transcriptional factor *AtMYB117*, a member of the R2R3 MYB gene family (subgroup 21) from *Arabidopsis* [8], [9].

To confirm that the *ctf* phenotype was conferred by the activated expression of *AtMYB117/LOF1*, the *AtMYB117/LOF1* cDNA was introduced into L*er* wild type plants under the control of the CaMV 35S promoter. Homozygous T2 lines of transgenic plants resembled *ctf* mutants with mild to severe phenotypes. Intensity of phenotype was correlated with the level of expression of the transgene, where plants with severe *ctf* phenotype had the highest level of *AtMYB117/LOF1* transcript (data not shown).

### Localization of the expression of *AtMYB117/LOF1* gene in L*er* plants

Lee *et al.* [10] analyzed the *AtMYB117/LOF1* spatial expression using the enhancer-trap line ET4016. GUS activity was restricted to adaxial boundary regions and the base of floral organs. To confirm whether the *ctf* phenotype was caused by overexpression of *AtMYB117/LOF1*, we examined the expression of *AtMYB117/LOF1* by RNA *in situ* hybridization in vegetative and reproductive tissues of L*er* and *ctf* plants.

**Figure 5:**
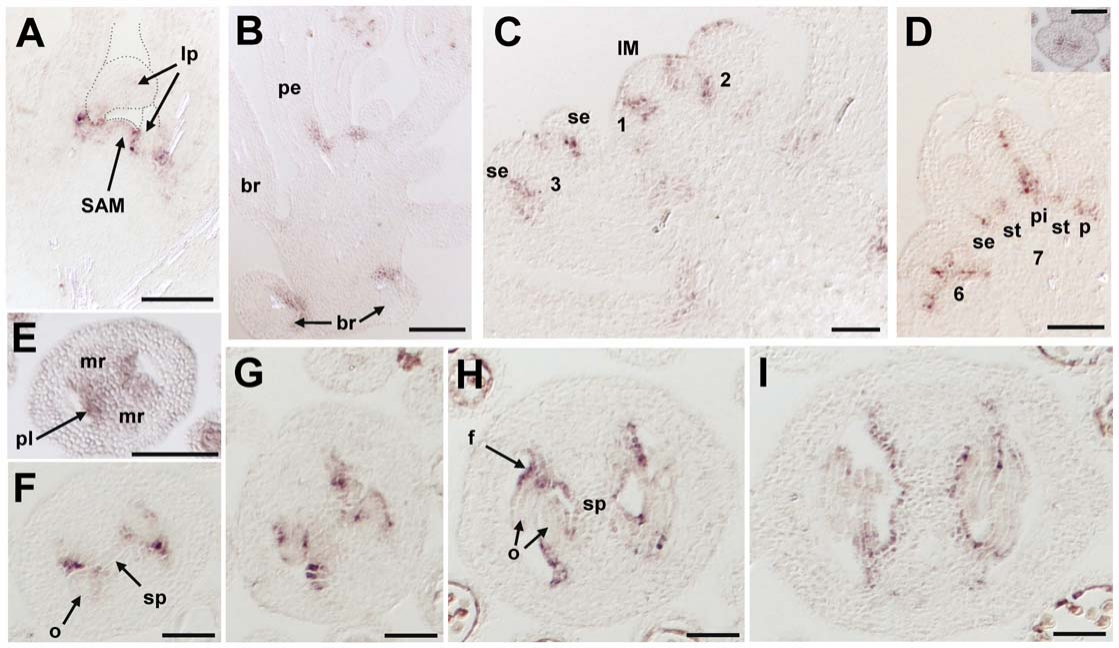
Localization of *AtMYB117/LOF1* gene expression in L*er* plants. Longitudinal section of apical shoot (seven-day old seedling) (**A**). Longitudinal section of inflorescence stem (**B**). Longitudinal section of inflorescence meristem and flower meristem at floral stages 1–3 (**C**). Longitudinal section of flower buds at floral stages 6 and 7 (**D**). Inset: transverse section of pistil primordium at stage 7. Transverse sections of developing pistil at floral stage 8 (**E**), early stage 9 (**F**), stage 9 (**G**), stage 10 (**H**) and late stage 10 (**I**). IM, inflorescence meristem; SAM, shoot apical meristem; br; bract; f, funiculus; lp, leaf primordia; mr, medial ridge; o, ovule primordium; p, petal; pe, petiole; pi, pistil; pl, placenta; se, sepal; sp, septum; st, stamen. Scale bars are 50 µm in **E–I** and inset in **D**; and 100 µm in **A–D**.

*AtMYB117/LOF1* transcripts were detected throughout all stages of development in L*er* plants. In young seedlings *AtMYB117/LOF1* transcripts were observed at the boundaries between the vegetative SAM and leaf primordia (Figure 5A). On longitudinal sections through the inflorescence stem, *AtMYB117/LOF1* transcripts were detected in a group of cells in the adaxial base of petioles and bracts (Figure 5B). Figure 5C shows floral meristems at stages 1–3 [4] arising on the flank of the IM. *AtMYB117/LOF1* was expressed at the boundaries between the IM and floral meristems. This signal was maintained and later observed in the adaxial-basal side of petioles (Figures 5B and C). Floral organ primordia are formed on the floral meristem where *AtMYB117/LOF1* transcripts were observed at the boundary between sepal primordia and the floral meristem at stage 3 (Figure 5C). *AtMYB117/LOF1* transcripts were detected at the boundaries between each of the four whorls (sepals, petals, stamens and pistil) beginning at floral stages 6 and 7 and continuing until stage 10 (Figure 5D). At stage 7, *AtMYB117/LOF1* transcripts were also detected at the inner of the medial ridges in the pistil (Figure 5D). At stage 9, expression was observed in the placenta (Figure 5E) and in the base of the ovule primordia (Figure 5F). Later, signal became restricted to the epidermis of the developing septum that is derived from the medial ridge and to the outer cell layers of the ovule funiculi (Figures 5G–I). *AtMYB117/LOF1* transcripts were not detected beyond stage 11.

### Localization of the expression of *AtMYB117/LOF1* gene in *ctf* plants

**Figure 6:**
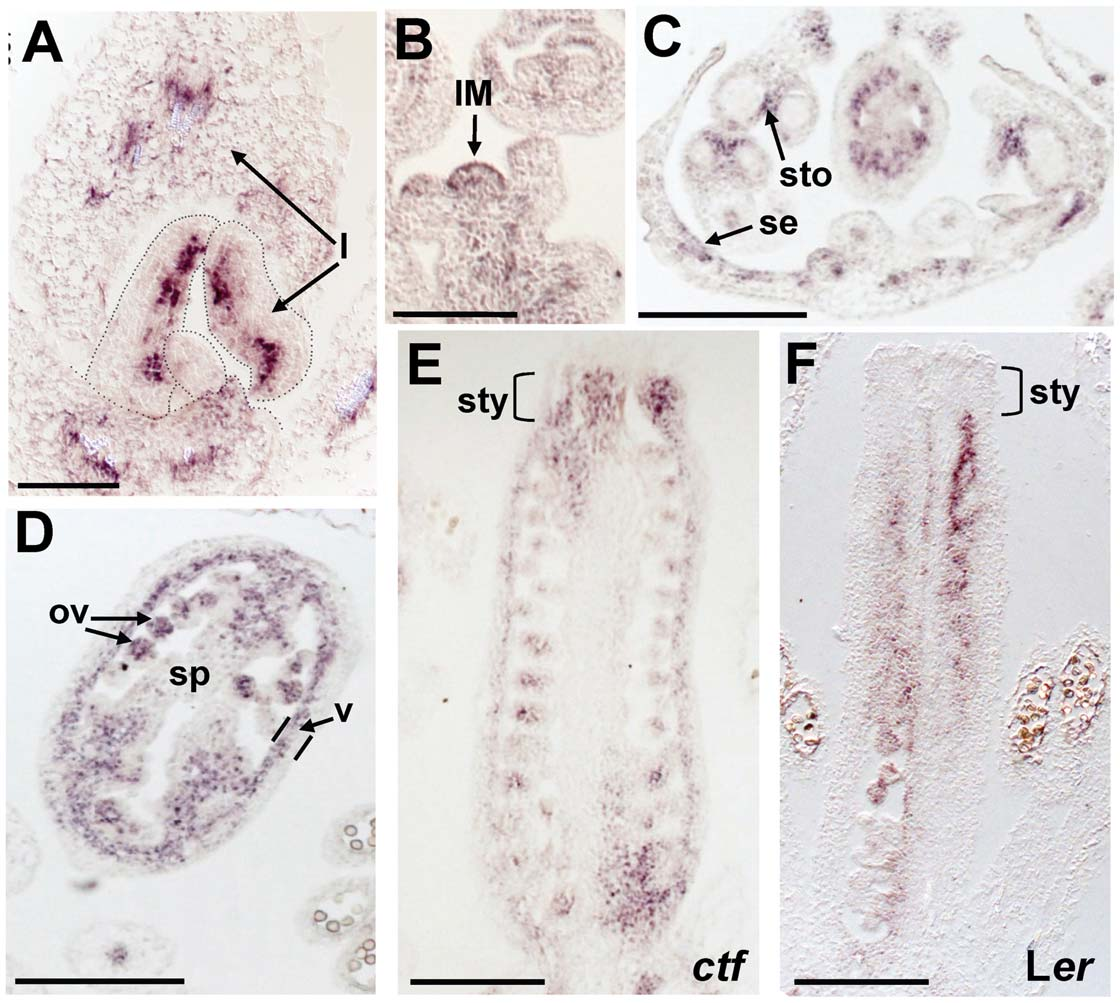
Localization of *AtMYB117/LOF1* gene expression in *ctf* mutant. Longitudinal section of apical shoot in seven-day old seedling (**A**). Longitudinal section of *ctf* inflorescence (**B**). Cross section of *ctf* flower at stage 8 (**C**). Cross section of a *ctf* pistil at stage 11 (**D**). Longitudinal sections of *ctf* pistil at stage 11 (**E**) and L*er* pistil at stage 10 (**F**). IM, inflorescence meristem; l, leaf; ov, ovule; se, sepal; sp, septum; sto, stomium; sty, style; v, valve. Scale bars are 100 µm in **A** and **B**; and 200 µm in **C–F**.

In *ctf* mutants, the expression of *AtMYB117/LOF1* was expanded spatially and temporally relative to wild type L*er* plants (Figure 6). The most remarkable differences were: 1) in seedlings, expression was observed in the adaxial side of hyponastic young leaves (Figure 6A); 2) in longitudinal sections of inflorescences, the signal filled the whole inflorescence meristem and stem (Figure 6B); 3) during flower development, expression was observed in the adaxial side of sepals and in cells around the stomium in stamens (Figure 6C); 4) in pistils, expression was localized to maturing ovules, the septum area close to the replum, the adaxial side (inner layers) of the valves (Figure 6D) and the style cells (Figures 6E, F). The signal was observed at least up to stage 13 (data not shown).

### Analysis of expression of pistil development genes in *ctf*

**Figure 7:**
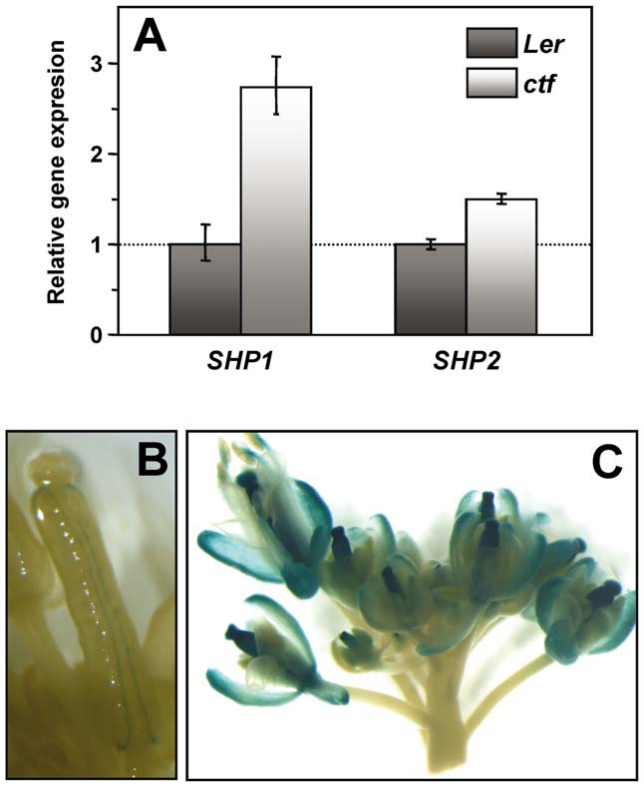
Analysis of *SHP1* and *SHP2* expression in L*er* and *ctf* inflorescences. (**A**) qRT-PCR analysis. Each experiment was carried out with three technical replicates and was repeated twice with similar results. Data expression normalized to *ACT8* (*At1g49240*) and relative to the expression of the L*er* inflorescence. Values are mean ± SD of a single experiment. *SHP2:GUS* expression pattern in L*er* pistil (**B**) and in *ctf* inflorescences (**C**); *SHP1* and *SHP2* show almost identical expression patterns.

The morphological characteristics of *ctf* fruit suggest that some of the genes that are known to control fruit development could be up or down-regulated in *ctf* mutants. To test this hypothesis, we analyzed the expression in inflorescences of *SHATTERPROOF1* (*SHP1*) and *SHP2*, that specify valve margin identity and promote ovule, stigma, style and medial tissue development [14], [15]; *REPLUMLESS* (*RPL*), that represses valve and valve margin development [16]; *FRUITFULL* (*FUL*), that is involved in valve cell development [17]; *BREVIPEDICELLUS* (*BP*), that was implicated in replum and valve margin development and *ASYMMETRIC LEAVES1* (*AS1*) and *AS2*, that regulates medio-lateral patterning of the fruit [18]. qRT-PCR analysis showed that only *SHP1* and *SHP2* transcript levels were remarkably increased in *ctf* (Figure 7A). *SHP2*-directed *GUS* expression, localized to valve margin in wild type L*er* plants (Figure 7B), was extended to the whole pistil and also to sepals in *ctf* fruit (Figure 7C). The increased expression in sepals correlated with the presence of carpelloid structures bearing ovules.

### *AtMYB117/LOF1* silencing by amiRNA

*lof1-1*, a T-DNA insertion mutant of *AtMYB117/LOF1*, had only a slight reduction of *AtMYB117/LOF1* transcript levels in inflorescence apex [10]. Therefore, as an alternative approach, we tried silencing *AtMYB117/LOF1* expression using an amiRNA strategy in L*er* and Col-0 ecotypes. *AtMYB117/LOF1* is closely related to *AtMYB105/LOF2*, and both function redundantly in boundary formation [10]. The possibility that *AtMYB117/LOF1* and *AtMYB105/LOF2* could also have redundant function in reproductive tissues and organs, prompted us to generate an amiRNA against both genes according to the described criteria [11]. amiRNA117/105 plants were tested for expression levels of both target genes in inflorescences by qRT-PCR analysis. Several transgenic lines were selected according to *AtMYB117/LOF1-AtMYB105/LOF2* expression levels and were brought to the homozygous state. Expression levels of these lines are shown in Figure S3. The amiRNA silenced both endogenous *AtMYB117/LOF1* and *AtMYB105/LOF2* expression with stronger effects observed in L*er* than in Col-0 ecotypes. However, none of the lines exhibited defects in inflorescence structure, and the observed phenotypes in the paraclade junctions in amiRNA117/105 plants were very similar to those described for *lof1-1* plants [10] (Figure S4). Funiculi with alterations in cellular morphology were also observed, but only for a few amiRNA117/105 plants (Figure S5).

## Discussion

We have isolated a gain-of-function mutant, named *ctf*, characterized by alterations in fruit morphology. It showed wrinkled valves, stigma with more prominent papillae, atypical style cells, wider replum, septum with an irregular structure and stacked seeds. The overexpressed gene was identified as *AtMYB117*, and overexpression of *AtMYB117* cDNA in *Arabidopsis* L*er* plants recapitulated the *ctf* phenotype. This gene, also named *LOF1*, was found to have a role in lateral organ separation and axillary meristem formation [10]. In *ctf* pistil and fruit our results demonstrate that *AtMYB117/LOF1* is expressed in the same tissues as in wild type fruit, but with higher intensity, and also in other tissues such as the style, inner layers of valves and mature ovules. Therefore, the alterations in fruit tissues might be explained by changes in the intensity of expression of *AtMYB117/LOF1*. CaMV 35S enhancers lead primarily to an enhancement of endogenous expression patterns, the resulting phenotype being a consequence of such an enhancement, as opposed to ectopic overexpression, reflecting the normal role of the activated gene [6].

A remarkable characteristic of *ctf* was the presence in sepals of ectopic carpelloid structures, including stigma, style, transmitting tissue and ovules. The identity of the ectopic tissues in *ctf* was confirmed by *GUS* activity directed by the *SPT* promoter, revealing the presence of transmitting tissue, not visible by cryo-SEM. Previous studies had shown that ectopic expression of *SHP1* and *SHP2*, involved in ovule, stigma, style and medial tissue development [15], is sufficient to induce the transformation of sepals into carpelloid organs bearing ovules [19], [20]. The formation of ectopic carpelloid structures in *ctf* sepals could be explained by the effect of the enhancement of *AtMYB117/LOF1* expression on the regulation of *SHP1* and *SHP2*. Increased expression of *SHP1* and *SHP2* and an extended localization of *SHP2:GUS* activity to the whole pistil and sepals was observed in *ctf* plants, while it is normally localized to the valve margins in wild type L*er* plants.

The *AtMYB117/LOF1* expression pattern obtained by RNA *in situ* hybridization extends the results obtained by Lee *et al.* [10], using an enhancer-trap line, in regard to the role of *AtMYB117/LOF1* in the establishment of organ boundaries. These authors detected *AtMYB117/LOF1* expression in the adaxial boundary regions between the SAM and lateral organs during vegetative development and between the inflorescence meristem and flower primordia. We also observed *AtMYB117/LOF1* transcripts in the adaxial boundary regions between each of the floral organs, in the boundaries of ovule primordia where they derive from the placenta, in the septum and in the region of ovule primordia that will give rise to the funiculus. To examine a possible role of *AtMYB117/LOF1* in the funiculus/ovule development we used amiRNA to obtain a knock-down mutant. Total silencing of *AtMYB117/LOF1* and *AtMYB105/LOF2* genes in the inflorescence was not achieved in any of the amiRNA117/105 lines. In spite of the low levels of *AtMYB117/LOF1* and *AtMYB105/LOF2* transcripts, the flowers and fruits did not present clear morphological changes. However, we did observe alterations in cellular morphology in the funiculi of some plants. This result, together with the observations mentioned above on *AtMYB117/LOF1* expression and the putative genetic relationship with *SHP1* and *2*, might suggest a functional role for *AtMYB117/LOF1* in funiculus/ovule development. Further studies will be needed to understand if the *AtMYB117/LOF1* gene is essential in ovule initiation and development and if *SHP1* and *SHP2* can be directly or indirectly regulated by the *AtMYB117/LOF1*.

*AtMYB117/LOF1* has a similar expression pattern to that of lateral organ boundary genes like *CUC2*, a member of the *NAC* family of transcription factors [21], and *JLO/LBD30*, a member of the *LBD* gene family [22]. Like *AtMYB117/LOF1*, these boundary genes are also expressed in the base of ovule primordia. *NAC*, *LBD*, and *GRAS* gene families have been identified as regulators involved in the definition of the boundaries of lateral organ regions [23]. Some LBD proteins can interact with other partners bearing MYB domains, such as AtAS2/LBD6 and AtASL4/AtLOB, which interact with AtAS1/AtMYB91. Therefore, AtMYB117/LOF1 might be a partner in a multiprotein complex, with a role in different steps and processes of plant development involving lateral organ formation. Fine-scale regulation of developmental processes could be modulated by differential regulation of one or more components, allowing for a great degree of plasticity.

### Concluding remarks

The characterization of the *ctf* mutant, the phenotype of amiRNA117/105 plants and the localization of *AtMYB117/LOF1* transcripts in boundary regions throughout different stages of development in *Arabidopsis*, including the initiation of ovule outgrowth, suggest that this gene is recruited in a variety of vegetative and reproductive developmental programs for the establishment of boundary regions and for funiculus/ovule development.

## Materials and Methods

### Plant materials and growth conditions

*Arabidopsis thaliana* Landsberg *erecta* (L*er*) and Columbia-0 (Col-0) seeds were obtained from the *Arabidopsis* Biological Resource Center (ABRC, www.biosci.ohio-state.edu). T-DNA insertion line, *lof1-1* (N525235) in the Col-0 ecotype, was obtained from NASC, the European *Arabidopsis* Stock Center (http://arabidopsis.info/), and homozygous mutants were selected by PCR-based genotyping. Sequences of genotyping primers used are available upon request. The *SHP2:GUS* reporter line [24] was kindly provided by Cristina Ferrándiz (IBMCP, Spain) and the *pSPT-6253:GUS* reporter line [13] by David Smyth (Monash University, Australia). *Arabidopsis* seeds were surface-sterilized, sowed in MS [25] plates and stratified at 4°C for 3 days in darkness. Seed were germinated by incubation in growth chambers at 22°C under a 16-h light/8-h dark photoperiod for one week, and seedlings were then transferred to soil and grown to maturity in the same growth conditions.

### Generation of activation-tagged transgenic plants

*Arabidopsis* transformation was performed as previously described using the activation-tagging binary vector plasmid pSKI015 [6]. This plasmid was introduced into GV3101 *Agrobacterium tumefaciens* cells and plants were transformed via the floral dip method [26]. Transformed seedlings were selected in plates of MS supplemented with 330 µM ammonium glufosinate (Fluka). The screening was done by morphological observations throughout the development of the transformed plants for phenotypes such as fruit length and shape. Homozygous and heterozygous plants were identified by genotype analysis by PCR (Table S1).

### Identification of the localization of the T-DNA insertions

The T-DNA insertion point in the *ctf* mutant was located by the plasmid rescue method [6]. Approximately, 1 µg of total genomic DNA was digested overnight with *Eco*RI. The digested DNA was ethanol precipitated, ligated overnight with T4 DNA ligase (New England Biolabs), and transformed into *E. coli* DH5α competent cells by electroporation. Ampicillin-resistant colonies were selected, and the rescued plasmids were purified, sequenced, and compared with the published *Arabidopsis* genome to determine T-DNA/genome junctions. qRT-PCR was carried out to test the expression level of genes around the predicted insertion point.

### Recapitulation analysis

The full-length *AtMYB117/LOF1* cDNA was isolated by PCR using gene-specific primers 117-5′ (CACCTTTCTCCCAACACTAACTCC) and 117-3′ (CCAAATTTAACCACTGTCGTTATCTG) and cloned into the pENTR™ Directional TOPO vector (Invitrogen). The resulting clones were confirmed by restriction analysis and sequencing. cDNA was transferred to the destination vector pK2GW7 under the control of the CaMV 35S promoter (Plant Systems Biology, VIB-Ghent University, Belgium) for over expression in plants [27]. Destination plasmid was transferred to *Agrobacterium tumefaciens* GV3301 strain to transform L*er* plants via the floral dip method. Transformed plants were selected on MS plates with 50 µM kanamycin. *AtMYB117/LOF1* expression level in transformed plants was measured by qRT-PCR with specific primers (see below).

### artificial microRNA (amiRNA) approach

To knock down both *AtMYB117/LOF1* and *AtMYB105/LOF2*, an amiRNA was generated as described [11]. The corresponding amiRNA was PCR amplified (Table S1) according to the protocol at Web MicroRNA Designer2 (WMD2; http://wmd2.weigelworld.org) and cloned into *Xba* I sites of the pFP101 vector system [28] under the control of the CaMV 35S promoter. Transgenic seeds were selected based on GFP signal under a Nikon fluorescence stereomicroscope and later genotyped with specific primers (Table S1).

### Gene expression analysis by qRT-PCR

Plant tissues were harvested, frozen with liquid N_2_, and stored at −80°C. Total RNA was extracted using the RNeasy Plant Mini Kit (Qiagen). Genomic DNA was eliminated with 50 units of DNaseI (Qiagen) for 15 min at room temperature. cDNA was synthesized using the SuperScript First-Strand Synthesis System for RT-PCR (Invitrogen). qRT-PCR analysis was carried out using the SYBR® GREEN PCR Master Mix (Applied Biosystems) in an ABI PRISM 7000 Sequence Detection System (Applied Biosystems) as described [29]. Primer sequences are indicated in the Table S1. In a single experiment, each sample was assayed in triplicate and the experiment was repeated twice, with similar results. *ACT8* (*At1g49240*) and *PP2A* Ser/Thr protein phosphatase 2A (*At1g13320*) were used as reference genes for normalization [30].

### *In situ* hybridization

Seedlings and inflorescences were embedded, sectioned and hybridised as described [31]. Two different templates for *AtMYB117/LOF1* were generated, a fragment of 223 bp containing the 5′ region of cDNA and an 847 bp fragment corresponding to the whole coding region of the *AtMYB117/LOF1* cDNA. The short probe was specific for the endogenous *AtMYB117/LOF1* gene and did not show any sequence similarity with *AtMYB105/LOF2*. Both fragments were cloned into the pGem-Teasy vector (Promega), and sense and antisense probes were synthesized using the corresponding SP6 and T7 RNA polymerases. After verifying that the two antisense probes gave identical results, we used the longer probe because it generated a more intense signal. Control experiments were performed with sense probes of *AtMYB117/LOF1* and no significant signal was detected (data not shown).

### Histological procedures

Plant tissues were fixed overnight in 4% (w/v) p-formaldehyde in 0.1 M sodium phosphate pH 7.2 with 0.05% (v/v) of Tween 20 at 4°C, dehydrated, and embedded in paraffin wax (Paraplast Plus) or Technovit 7100 resin as described [32]. Samples embedded in paraffin were sectioned on a rotatory microtome at 8 µm and stained with 1% Alcian blue 8GX and 1% Safranine O in 50% ethanol or 0.02% Toluidine blue. Tissues embedded in resin were sectioned in a Reichert Jung Ultracut E microtome at 3 µm and stained with 1% Alcian blue 8GX. Pistils from L*er* and *ctf* plants were harvested at anthesis, fixed, dehydrated and cleared with chloral hydrate according to [33]. Images were captured using a microscope Eclipse E600 (Nikon) equipped with Nomarski interference optics.

### GUS staining

To monitor *SPT* and *SHP2* expression, the *pSPT-6253:GUS* and *SHP2:GUS* reporter lines were crossed to *ctf* mutant. T2 segregants were genotyped for *ctf* and homozygous plants were stained for GUS activity. Inflorescences were fixed 30 min in 90% acetone, washed in staining buffer (50 mM sodium phosphate pH 7.0, 10 mM potassium ferricyanide, 10 mM potassium hexacyanoferrate, and 0.2% Triton X-100), and incubated overnight at 37°C in staining buffer supplemented with 0.1 mM X-GlcA (5-bromo-4-chloro-3-indolyl-b-D-glucuronide cyclohexylammonium). Samples were dehydrated to 70% (v/v) ethanol and observed in a stereoscopic microscope (Leica MZ16) and an Eclipse 600 microscope (Nikon) equipped with Nomarski interference optics.

### cryo-SEM

Samples were harvested, mounted on SEM stubs attached to the specimen holder of a CT-1000C cryo-transfer system (Oxford Instruments) and frozen in liquid N_2_. The frozen specimens were transferred to the cryo-stage of a JEOL JSM-5410 scanning electron microscope, sublimated by controlled heating at −85°C and sputter coated with a thin film of gold. Finally, samples were observed at incident electron energy of 10 keV.

## Supporting Information

Figure S1 **Localization of** ***SPATULA*** **gene expression in** ***ctf*** **flowers.** GUS expression driven by the *SPT* promoter in L*er* (**A**) and *ctf* (**B**) flowers. Enlargement of the boxed carpelloid structure observed by Nomarski technique (**C**). pi, pistil, ov, ovule; se, sepal; tt, transmitting tissue. Scale bars are 100 µm.
(TIF)

Figure S2 **Localization of the insertion point of the T-DNA in the** ***ctf*** **mutant.** (**A**) Detail of the genome region, showing the genes up- and down-stream of the insertion; overexpressed gene (*At1g26780 AtMYB117/LOF1*) in the *ctf* mutant is encircled. (**B**) Scheme of the activation tagging T-DNA and position of the restriction sites used for plasmid rescue.
(TIF)

Figure S3 ***AtMYB117/LOF1*** **and** ***AtMYB105/LOF2*** **expression analysis by qRT-PCR.** Relative gene expression in inflorescence of amiRNA117/105 lines in ecotypes L*er* and Col-0. Each experiment was carried out with three technical replicates and was repeated twice with similar results. Data (expression normalized to *ACT8* and relative to the expression of the inflorescence of L*er* or Col- 0 in each case) are mean ± SD of a single experiment.
(TIF)

Figure S4 **Phenotypes of** ***lof1-1*** **and amiRNA117/105 plants.** Col-0 and *lof1-1* plants (**A**). L*er* and amiRNA117/105 (line 12) plants (**B**). Note fused paraclade junctions in both mutant plants (red arrows).
(TIF)

Figure S5 **Morphological characteristics of funiculi in amiRNA117/105 fruits.** Cryo-scanning electron micrographs of funiculi of L*er* fruits (**A**) and amiRNA117/105 fruits (**B–D**) at late stage 17. Scale bar is 50 µm.
(TIF)

Table S1 Primers used in this work.
(DOC)
