# Supplementary figures and images for: Characterization of constricted fruit (ctf) Mutant Uncovers a Role for AtMYB117/LOF1 in Ovule and Fruit Development in Arabidopsis thaliana
Source: PLoS One. 2011 Apr 13;6(4):e18760. doi: 10.1371/journal.pone.0018760 (PMC3076444; doi:10.1371/journal.pone.0018760)

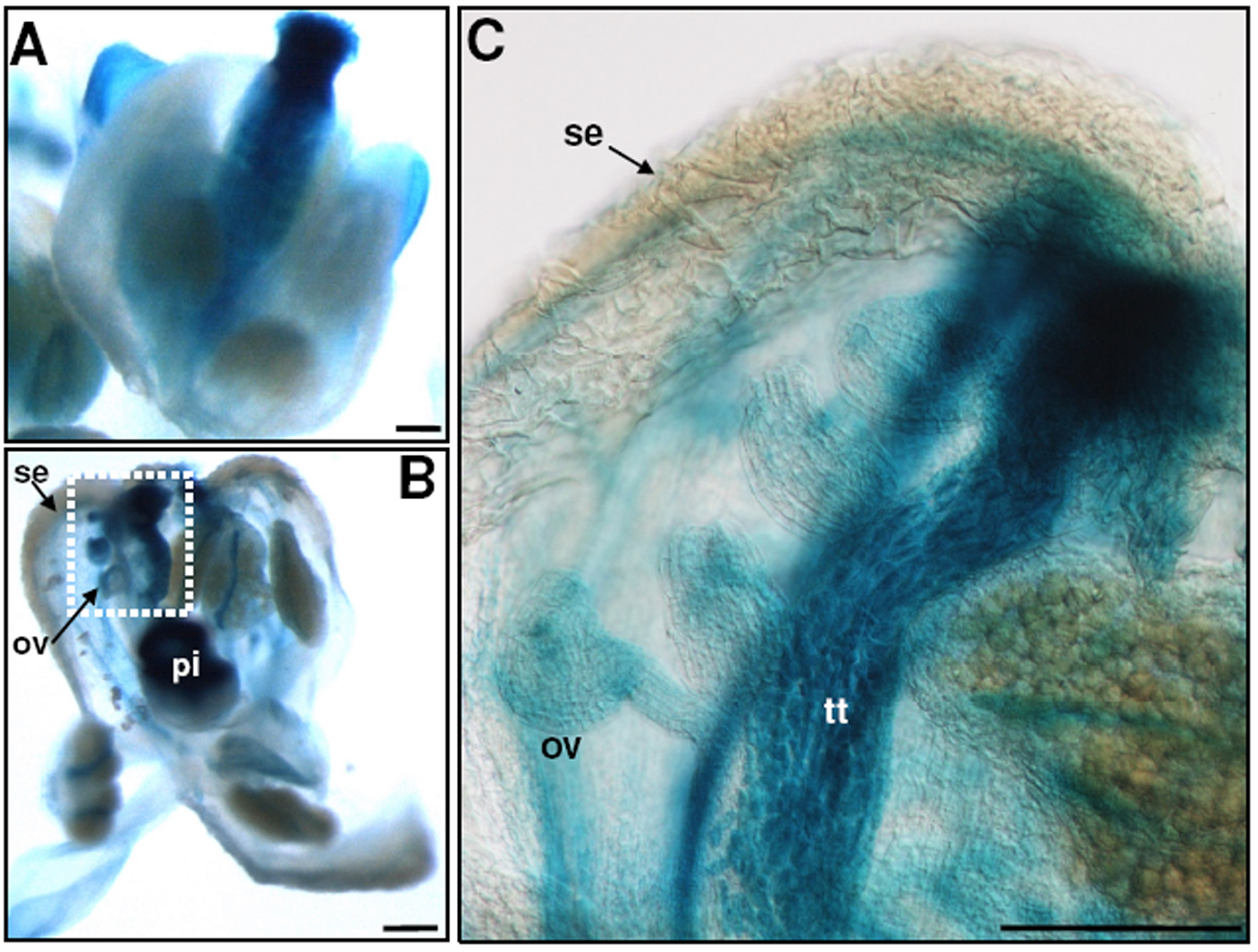

Supplement: Figure S1 — Localization of SPATULA gene expression in ctf flowers. GUS expression driven by the SPT promoter in Ler (A) and ctf (B) flowers. Enlargement of the boxed carpelloid structure observed by Nomarski technique (C). pi, pistil, ov, ovule; se, sepal; tt, transmitting tissue. Scale bars are 100 µm. (TIF) [file pone.0018760.s001.tif]

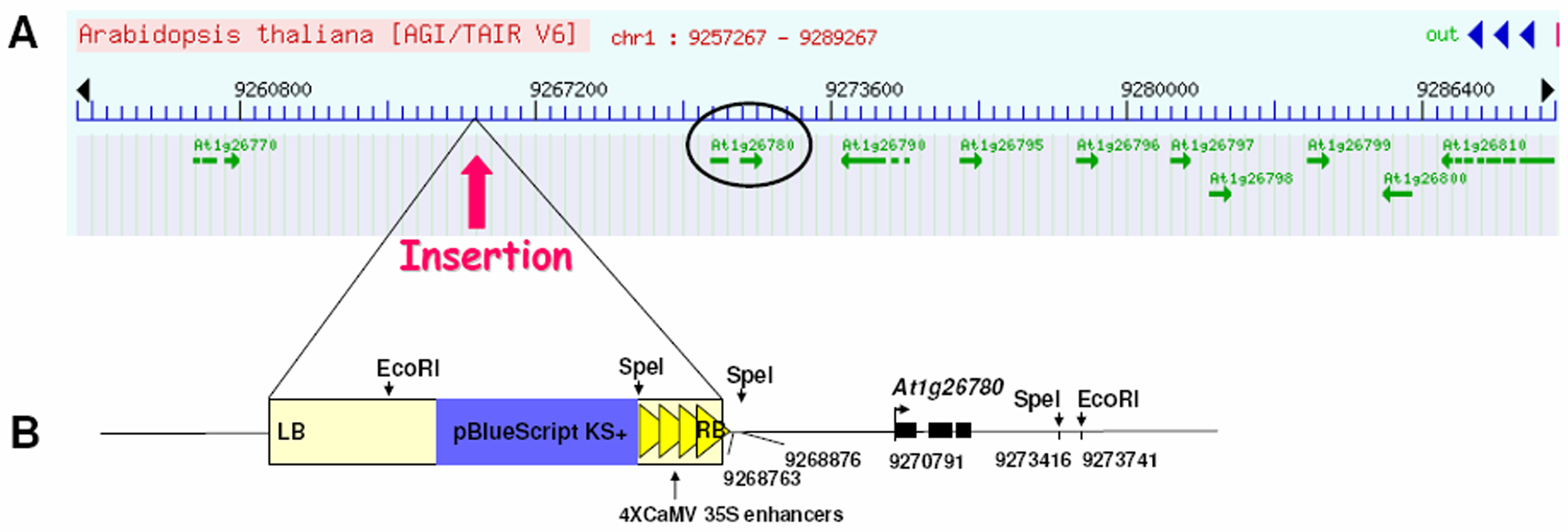

Supplement: Figure S2 — Localization of the insertion point of the T-DNA in the ctf mutant. (A) Detail of the genome region, showing the genes up- and down-stream of the insertion; overexpressed gene (At1g26780 AtMYB117/LOF1) in the ctf mutant is encircled. (B) Scheme of the activation tagging T-DNA and position of the restriction sites used for plasmid rescue. (TIF) [file pone.0018760.s002.tif]

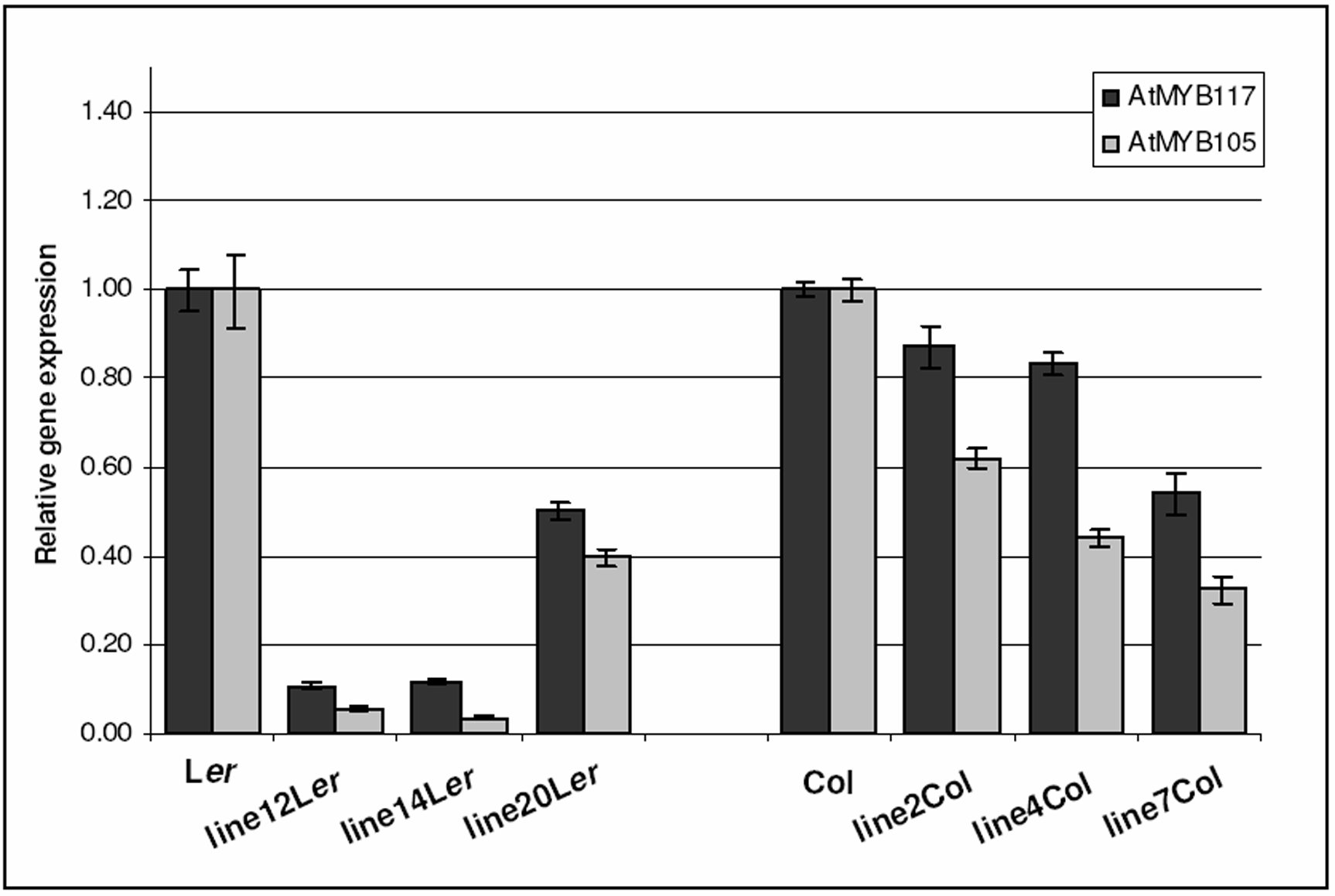

Supplement: Figure S3 — AtMYB117/LOF1 and AtMYB105/LOF2 expression analysis by qRT-PCR. Relative gene expression in inflorescence of amiRNA117/105 lines in ecotypes Ler and Col-0. Each experiment was carried out with three technical replicates and was repeated twice with similar results. Data (expression normalized to ACT8 and relative to the expression of the inflorescence of Ler or Col- 0 in each case) are mean ± SD of a single experiment. (TIF) [file pone.0018760.s003.tif]

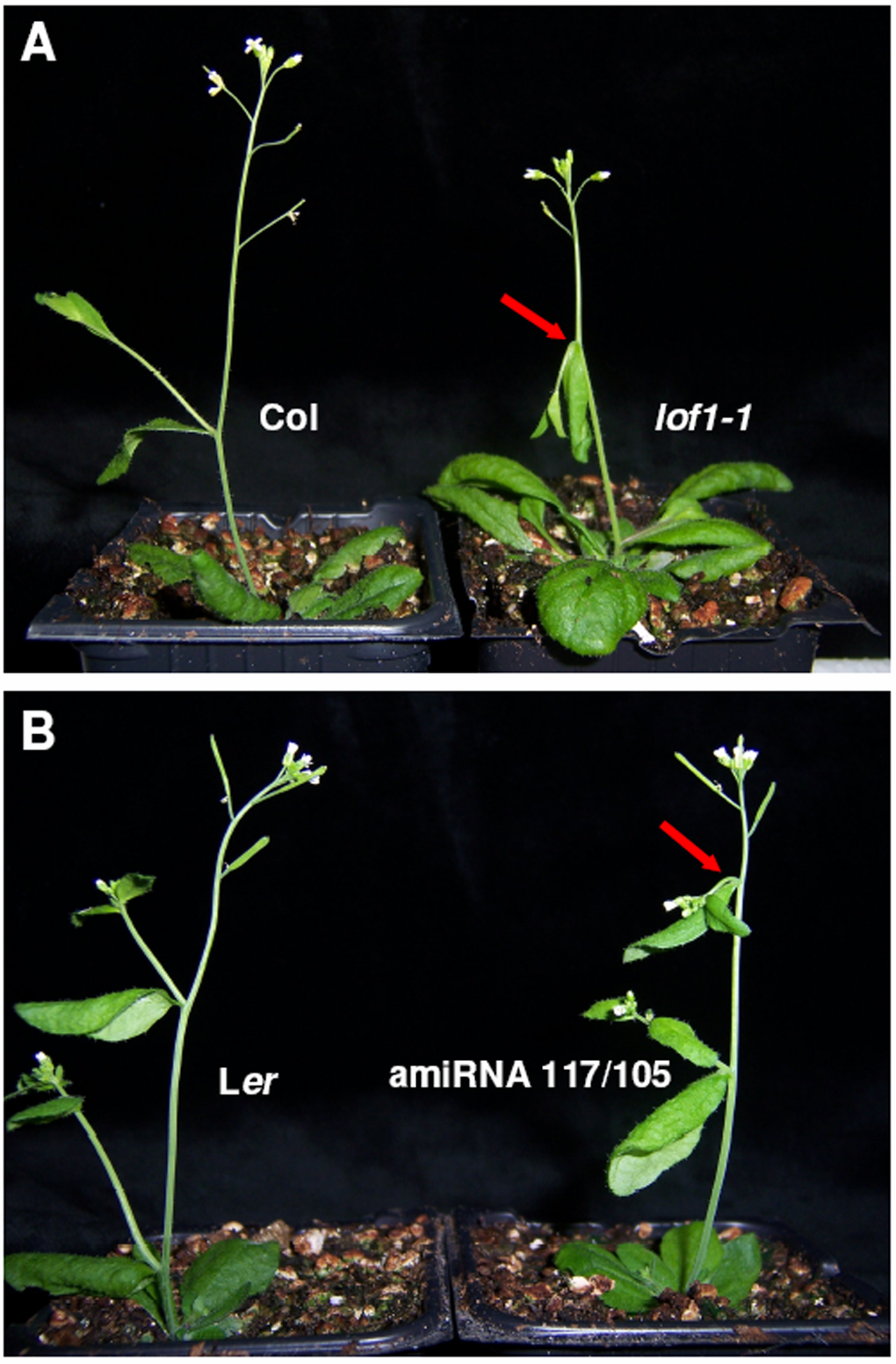

Supplement: Figure S4 — Phenotypes of lof1-1 and amiRNA117/105 plants. Col-0 and lof1-1 plants (A). Ler and amiRNA117/105 (line 12) plants (B). Note fused paraclade junctions in both mutant plants (red arrows). (TIF) [file pone.0018760.s004.tif]

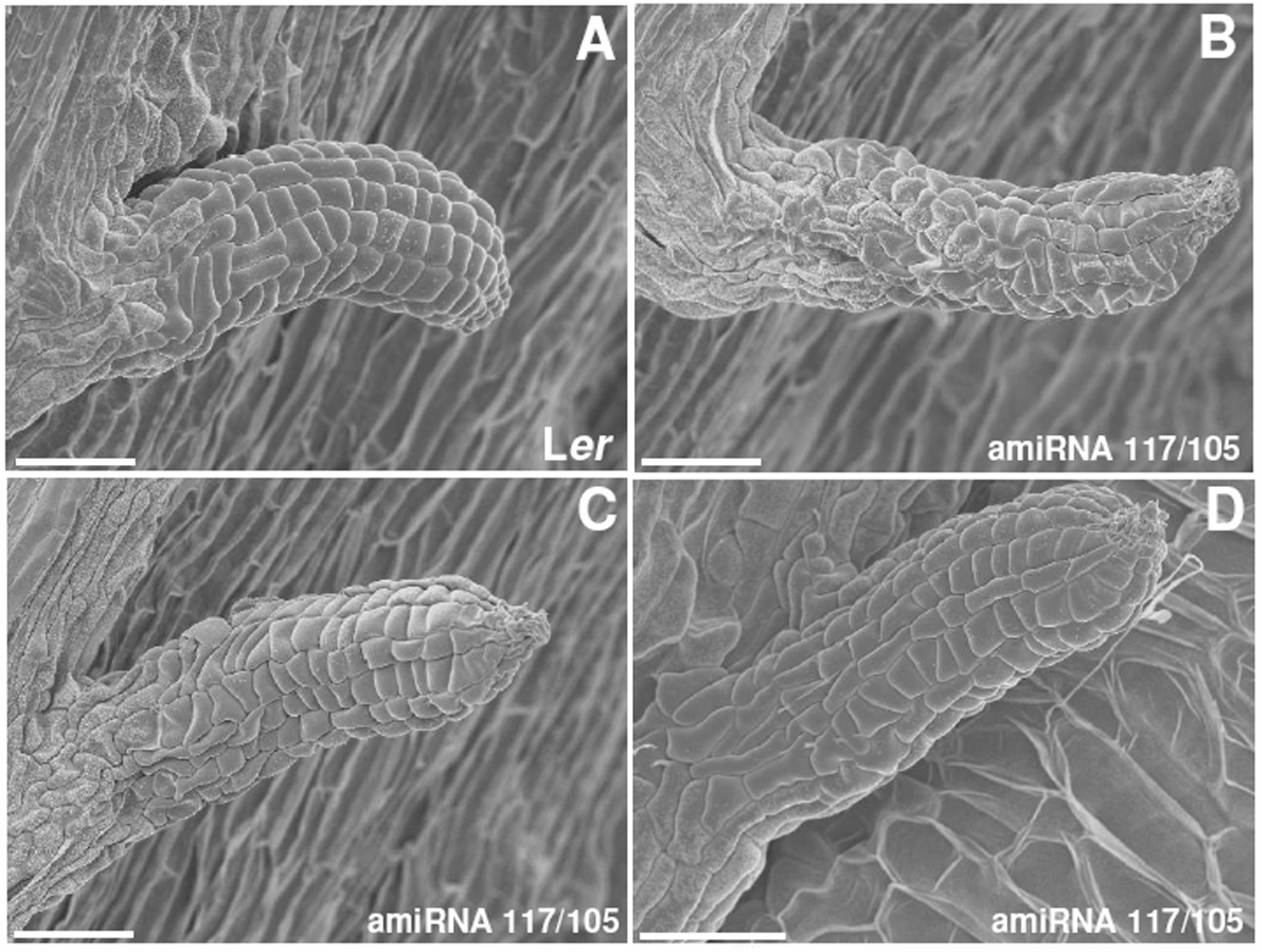

Supplement: Figure S5 — Morphological characteristics of funiculi in amiRNA117/105 fruits. Cryo-scanning electron micrographs of funiculi of Ler fruits (A) and amiRNA117/105 fruits (B–D) at late stage 17. Scale bar is 50 µm. (TIF) [file pone.0018760.s005.tif]
