# Supplementary material for: Characterization of constricted fruit (ctf) Mutant Uncovers a Role for AtMYB117/LOF1 in Ovule and Fruit Development in Arabidopsis thaliana
Source: PLoS One. 2011 Apr 13;6(4):e18760. doi: 10.1371/journal.pone.0018760 (PMC3076444; doi:10.1371/journal.pone.0018760)
Supplement: Table S1 — Primers used in this work. (DOC) [file pone.0018760.s006.doc]

**Table S1.** Primers used in this work.

| **PRIMER** | **SEQUENCE** | **PURPOSE** |
| --- | --- | --- |
| ACTIN 8 F | AGTGGTCGTACAACCGGTATTGT | Quantitative PCR Actin 8 (constitutive gene) |
| ACTIN 8 R | GAGGATAGCATGTGGAAGTGAGAA | Quantitative PCR Actin 8 (constitutive gene) |
| PP2A F | TAACGTGGCCAAAATGATGC | Quantitative PCR PP2A (constitutive gene) |
| PP2A R | GTTCTCCACAACCGCTTGGT | Quantitative PCR PP2A (constitutive gene) |
| At1g26770F | GATCAACGCTCACGCCACTT | Quantitative PCR At1g26770 (Plasmid rescue) |
| At1g26770R | ATTGTGCCGGAAGCATCAC | Quantitative PCR At1g26770 (Plasmid rescue) |
| At1g26780F | GCTGATGCAAGCACATAGGC | Quantitative PCR of AtMYB117 |
| At1g26780R | CTCGCAATCATTGCCCATT | Quantitative PCR of AtMYB117 |
| At1g26760F | AGCTGTGGTGAAGACTGGTGG | Quantitative PCR At1g26760 (Plasmid rescue) |
| At1g26760R | CACCGGGTCTAACACTTGCAG | Quantitative PCR At1g26760 (Plasmid rescue) |
| At1g26790F | GGGTTACTTCCTCCCCAAGC | Quantitative PCR At1g26790 (Plasmid rescue) |
| At1g26790R | TTGGTAAGGCCAAGGAGGC | Quantitative PCR At1g26790 (Plasmid rescue) |
| At1g26795F | CAGGTTTCGTGTCAATCTCCG | Quantitative PCR At1g26795 (Plasmid rescue) |
| At1g26795R | GGCCACTCGAAACTGCAAGT | Quantitative PCR At1g26795 (Plasmid rescue) |
| Myb105 F | TGGTGGATATGTTCGAAACCC | Quantitative PCR of AtMYB105 |
| Myb105 R | TCCATGCAATGTAATCGCCA | Quantitative PCR of AtMYB105 |
| 1miR-MYBs-s | GATTAAAGCCTATGAGCGTGCATTCTC TCT TTTGTATTCC | amiRNA117-105 construct  amiRNA117-105 genotyping |
| 2miR-MYBs-a | GAATGCACGCTCATAGGCTTTAATCAA AGA GAATCAATGA | amiRNA117-105 construct |
| 3miR- MYBs*s | GAATACACGCTCATACGCTTTATTCAC AGG TCGTGATAT | amiRNA117-105 construct |
| 4miR-MYBs*a | GAATAAAGCGTATGAGCGTGTATTCTA CAT ATATATTCCT | amiRNA117-105 construct |
| T24P13-68853 | GTACTAGATTTTCGATTCTTTCGTC | *ctf* genotyping |
| T24P13-68334 | GGGCTATGGGGTCAAAGAATGGTCGAG | *ctf* genotyping |
| pSKI15-RB1 | CCAACATGGTGGAGCACGACACTCTCGTCT | *ctf* genotyping |
| FVS 267 | TACATGCTTAACGTAATTCAACAGA | amiRNA117/105 genotyping |
| SHP1 F | CAA AGA TAG CCG AAG GCG C | Quantitative PCR of SHP1 |
| SHP1 R | CAC TCG ATT CCT GCT GGT CC | Quantitative PCR of SHP1 |
| SHP2 F | TGA CGC TGA GGT TGC TCT TG | Quantitative PCR of SHP2 |
| SHP2 R | TCG TAG AGA CGG CCT CGA GT | Quantitative PCR of SHP2 |
